# Organizational and administrative requirements of NGOs for environmental crisis response: a field study of civil associations

**DOI:** 10.3389/fpubh.2026.1956771

**Published:** 2026-10-06

**Authors:** Shimaa Abdelaziz, Abdelhamed Bakry, Edris Alam, Guoqing Shi

**Affiliations:** 1 School of Public Administration, Hohai University, Nanjing, China; 2 Faculty of Social Work, Aswan University, Aswan, Egypt; 3 Faculty of Resilience, Rabdan Academy, Abu Dhabi, United Arab Emirates; 4 Department of Geography and Environmental Studies, University of Chittagong, Chittagong, Bangladesh

**Keywords:** civil society organizations, crisis management, Egypt, environmental crisis response, NGO capacity, organization theory

## Abstract

Environmental crises are increasing in frequency and severity across Egypt, yet the organizational requirements of civil society organizations for effective response to these events remain insufficiently documented. This cross-sectional census survey assessed the administrative, human resource, financial, and legislative requirements necessary for effective environmental crisis response among non-governmental organizations (NGOs) in Aswan, Egypt. Data were collected from 109 board members and executive directors representing 13 NGOs using a structured 16-item questionnaire. Descriptive statistics, independent-samples t-tests, one-way analysis of variance with post-hoc comparisons, Pearson correlation analysis, and multiple regression were used to analyze the data. The overall level of organizational requirements among NGOs in facing environmental crises was high (M = 3.84, SD = 0.49). Administrative requirements received the highest mean score (M = 4.06, SD = 0.67), whereas financial requirements received the lowest, although they remained within the high range (M = 3.75, SD = 0.65). All four organizational requirements were positively correlated, with the strongest association observed between financial and legislative requirements (r = 0.926, *p* < 0.001). Differences were identified based on selected personal and organizational characteristics, with organizational age showing the most consistent association with NGOs’ capacity for environmental crisis response. The regression model was statistically significant and explained 35.6% of the variance in the overall score (R^2^ = 0.356). Organizational age was the only significant independent predictor in the model (B = 3.86, *p* < 0.001), whereas individual board-member experience and gender were not significant after adjustment. These findings indicate that organizational maturity is an important correlate of NGO capacity for environmental crisis response. Capacity-building programs, donor support, and institutional partnerships should therefore give particular attention to newer NGOs while strengthening financial contingency planning and formal crisis-management procedures across organizations.

## 1 Introduction

The world is confronted with accelerating and interconnected environmental crises, including climate change, biodiversity loss, pollution, and resource depletion, that operate at a global scale and stem largely from human-driven pressures. Climate change contributes to escalating extremes, ecosystem instability, and global economic risks (1, 2), while biodiversity loss is recognised as one of the three major emergencies threatening human and ecological well-being (3). Pollution from agricultural waste, industrial activities, and toxins increases greenhouse gas emissions and further degrades natural systems (4, 5), rapid resource depletion intensifies environmental degradation and water scarcity worldwide (6). These crises collectively underscore the need for transformative, globally coordinated environmental governance and sustainability education to address systemic risks in the Anthropocene (7).

When these global pressures translate into national contexts such as Egypt, they manifest through severe water scarcity linked to dependence on the Nile and rising evapotranspiration (8, 9), increasing irrigation demands that reduce crop productivity and stress key agricultural systems such as wheat and potatoes (10). Pollution poses additional threats, with studies documenting degraded water quality and heavy metal contamination in major ecosystems such as Lake Burullus and Lake Idku (11). Egypt also experiences land degradation and desertification that undermine soil health and food security (12), while climate-related hazards including extreme rainfall, flash floods, sea-level rise, and coastal ecosystem risks in the Red Sea intensify national vulnerability (13).

These compounding pressures underscore the need for organizations capable of responding swiftly and effectively. Among the actors positioned to meet this need, civil society organizations, and non-governmental organizations (NGOs) in particular, act as essential first-responders because they are rooted in communities, mobilize quickly, and reach vulnerable groups often overlooked by formal systems (14). NGOs have also been found to provide frontline assistance during crises through localized rescue, relief, and support services, demonstrating agility and trust-based access that governments frequently lack (15). This aligns with the Sendai Framework, which calls for people-centered, community-empowered disaster risk reduction and emphasizes shared responsibility between state and non-state actors (16). NGOs also advance SDG 11 by strengthening local resilience and community preparedness, and SDG 13 by leading grassroots climate action, advocacy, and adaptation initiatives (17). Across crises, from pandemics to natural disasters, NGOs repeatedly fill operational gaps, coordinate assistance, and sustain community resilience, making them indispensable components of effective crisis governance and rapid response systems (18).

Despite the growing frequency and severity of environmental crises in Egypt, and despite NGOs being widely regarded as essential first responders, questions remain regarding whether these organizations possess the organizational and administrative capacities needed to respond effectively when such crises occur. Weak internal structures, limited financial reserves, insufficient trained personnel, or unclear legal mandates may each constrain an NGO’s ability to act swiftly and effectively during an environmental crisis, yet the extent to which Egyptian NGOs are organizationally prepared for this role remains insufficiently documented. This raises the central problem addressed by the present study: to what extent do NGOs in Egypt possess the organizational and administrative requirements necessary to respond effectively to environmental crises, how are these requirements interrelated, and to what extent does their availability vary according to the personal and organizational characteristics of those responsible for them?

Accordingly, this study aims to examine the organizational and administrative requirements necessary for NGOs to respond effectively to environmental crises, to assess the extent to which these requirements are available among the NGOs under study, to explore the relationships among the different organizational requirements, and to identify whether their availability differs according to respondents’ personal and organizational characteristics, including which of these characteristics contributes most strongly to explaining the overall organizational requirements score. Based on the study objectives and research questions, the following null hypotheses were formulated:

*H1:* There are no statistically significant differences in organizational requirements according to respondents’ gender.

*H2:* There is no statistically significant effect of age on organizational requirements.

*H3:* There is no statistically significant effect of educational qualification on organizational requirements.

*H4:* There is no statistically significant effect of organizational role on organizational requirements.

*H5:* There is no statistically significant effect of years of experience on organizational requirements.

*H6:* There is no statistically significant effect of organizational age on organizational requirements.

## 2 Literature review

### 2.1 The role of NGOs in environmental crisis response

Research consistently shows that NGOs play essential and multifaceted roles in environmental crisis response, acting as rapid responders, long-term resilience builders, and governance intermediaries. Across disaster contexts such as floods, earthquakes, and wildfires, NGOs provide immediate relief, including food, shelter, and medical support, often operating faster than government agencies due to fewer bureaucratic constraints (19–23). Beyond emergency relief, NGOs increasingly engage in disaster risk reduction by offering community preparedness training, raising awareness, and promoting mitigation frameworks, although their formal integration into national DRR systems remains uneven (24–27). In climate-vulnerable regions, NGOs strengthen adaptation and resilience, acting as knowledge brokers, mobilizing resources, and supporting marginalised groups, though challenges such as elite capture and institutional barriers can limit their effectiveness (28–32). Their governance roles extend to policy advocacy, environmental monitoring, and representing community interests, helping democratize decision-making and enhance public participation, particularly where state capacity is weak or public trust is low (33–36). NGOs also foster community resilience by leveraging social networks, empowering local actors, and enabling collective action, which is critical in fragmented governance environments (24, 37). However, their effectiveness is often constrained by coordination failures, resource shortages, donor dependence, regulatory restrictions, and the risk of creating community dependency on external aid (38–41). Emerging trends highlight NGOs’ increasing use of digital communication tools for environmental advocacy and multi-stakeholder governance models involving governments, businesses, and research institutions (42).

### 2.2 Organizational and administrative requirements for crisis response

Organizational and administrative requirements for crisis response center on developing systematic, proactive, and structurally embedded capabilities that enable institutions to anticipate, withstand, and adapt to disruptive events. Across the literature, crisis-prepared organizations implement systematic and systemic preparedness programs, combining structured planning, clear decision-making processes, and organization-wide coordination mechanisms to ensure timely and informed action under uncertainty (43). Effective preparedness requires institutional resilience, defined as the capacity of government and organizational agencies to prevent crises, sustain essential services during disruptions, and support community recovery afterward (44). Multiple studies emphasize the necessity of formal crisis management plans that are regularly updated, supported by designated crisis teams, pre-drafted communication messages, and annual exercises to test and refine preparedness procedures (45). Organizational structure and culture play a decisive role: rigid functional structures may fragment crisis planning, while flexible, cross-functional structures enhance coordination, rapid response, and organizational learning (46, 47). Crisis-prepared organizations also establish early warning and crisis-sensing mechanisms, enabling the detection and diagnosis of emerging threats before they escalate (48). Administrative preparedness further depends on resource planning, including stockpiling essentials, establishing communication systems, and ensuring staff training through simulations and drills (49, 50). Governance literature stresses the importance of multi-level coordination, robust institutional arrangements, and inter-agency cooperation, particularly where crises cross administrative boundaries or require joint task-force operations (51, 52). Maintaining institutional memory, routine leadership briefings, and embedding preparedness requirements into statutory planning cycles helps avoid knowledge erosion and supports continuity across administrative turnover (53). Additionally, secure communication networks and reliable information flows are essential administrative components, ensuring real-time coordination during crises (54). Finally, crisis preparedness is strengthened through organizational learning processes, emphasizing involvement of relevant actors, clear preparedness goals, and structured review of plans, forming an iterative learning cycle that enhances long-term resilience (55).

### 2.3 NGOs’ capacity in the Arab regional context

NGO capacity in the Arab regional context is shaped by a complex interplay of governance environments, political constraints, resource limitations, and evolving development agendas. Research on the region highlights that Arab NGOs operate within highly varied socio-political settings, ranging from restrictive regulatory environments, such as in Jordan where governance and accountability challenges persist in the post-Arab Spring era (56). In Egypt specifically, the regulatory relationship between the state and civil society has remained a defining feature of the NGO sector, particularly following the 2017 and 2019 NGO laws, which shaped both the operational constraints and the public framing of civil society organizations (57, 58). To conflict-affected contexts like Palestine, where NGOs face structural crises driven by conditional donor funding and state pressures, despite their vital roles in democratization, resilience, and rights promotion (59). Across the Middle East, NGOs and broader civil society organizations contribute significantly to addressing developmental disparities, providing services in sectors such as education, health, human rights, and environmental sustainability, while navigating political restrictions, financial dependency, and operational barriers particularly acute in conflict zones such as Yemen, Lebanon, and Syria (60). Capacity-building literature shows that the Arab nonprofit sector remains under-researched, with only limited studies documenting institutional development, localized volunteering, or the role of religious endowments as distinctive features of organizational capacity in the region (61). Human rights organizations in Palestine similarly face challenges tied to weak human rights cultures and institutional barriers, which constrain their ability to meet socio-economic and political rights obligations (62). Conflict settings particularly strain NGOs’ administrative and operational capacities: studies in Yemen document bureaucratic barriers, coordination difficulties with UN partners, and limited support for local NGO capacity development, all of which undermine effective humanitarian delivery (63). Broader regional analyses also show that civil society engagement in service delivery carries mixed implications for political change, at times bolstering community legitimacy and mobilization, while in other cases reinforcing elite structures that limit transformation (64). Despite these constraints, Arab NGOs often fill critical governance gaps, especially in areas where state capacity is weak, providing essential welfare services and contributing to social protection systems, as observed across the Arab world’s diverse governance contexts (65). While the reviewed literature confirms the importance of NGOs in crisis response and outlines the general organizational requirements that enable effective response, few studies empirically examine these requirements as specific, measurable requirements within Arab NGOs, particularly in the context of environmental crises in Egypt. This study addresses that gap by drawing on Organization Theory to empirically examine the administrative, human resource, financial, and legislative requirements of Egyptian NGOs in facing environmental crises.

## 3 Theoretical framework

Organizational Theory is the body of scholarship that examines how formal organizations structure authority, divide labor, and coordinate activity in order to achieve their goals efficiently and rationally (66). Max Weber is widely credited as the founder of this field through his theory of bureaucracy, which he developed as part of his broader analysis of rationalization and legal-rational authority in modern society. Weber identified bureaucracy as the most technically efficient form of organization, characterized by a fixed hierarchy of authority, formal division of labor based on specialization, impersonal and rule-governed decision-making, and appointment based on technical competence rather than personal favor (67). Organization Theory has been applied by researchers in varied ways to explain how organizations structure themselves and respond to internal and external demands. Built directly (68) on Organization Theory to develop the “Organization Theory for Implementation Science” (OTIS) framework, integrating multiple organizational sub-theories to explain how organizational characteristics shape the successful implementation of new practices within institutions. Walton (69) applied Organization Theory empirically by conducting a meta-analysis of nearly four decades of research testing Weber's model of bureaucratic control, finding that its core structural variables remain strongly and consistently associated with one another, confirming the model's continued relevance to modern organizations. Nderi (68) applied Organization Theory’s contingency perspective to examine how organizations align their internal structure with the demands of their external environment, arguing that organizational effectiveness depends on the fit between structural design and environmental volatility. Organization Theory frames each of the study’s four organizational requirements as a distinct structural component of organizational functioning. Administrative requirements reflect the theory’s emphasis on formal coordination and structured decision-making; human resource requirements reflect its concern with allocating roles according to competence; financial requirements reflect the organization’s dependence on securing external resources for continuity; and legislative requirements reflect its attention to compliance with external regulatory frameworks. This framing allows the study’s findings to be interpreted not merely as isolated scores, but as indicators of organizational structural adequacy. The selection of these four organizational requirements was guided by Organization Theory and the contingency perspective (70). Administrative and human resource requirements reflect internal structural mechanisms related to coordination, decision-making, and competencies (71), while financial and legislative requirements represent the organization’s adaptation to resource and regulatory environments (72). Together, these requirements capture key structural conditions that influence NGOs’ capacity to respond effectively to environmental crises (73).

## 4 Methodology

### 4.1 Research design

This study adopts a descriptive survey design within a post-positivist, deductive framework. Descriptive research enables the systematic collection, analysis, and interpretation of data to accurately characterize phenomena as they exist in their natural context (74). This design is particularly suited to the present study, which aims to identify and assess the organizational and administrative requirements of NGOs in confronting environmental crises, a subject that demands precise mapping of current capacities and existing gaps across the different types of organizational requirements.

### 4.2 Population, sampling and study setting

Given the bounded and identifiable nature of the target population, a total population census was employed rather than a probability sample. The study population comprised all board members of environmentally engaged NGOs officially registered with the Aswan Directorate of Social Solidarity and operating within the administrative boundaries of Aswan city, Egypt. Organizations were included if they were classified as active and operationally engaged by the Directorate, with demonstrated activities within their stated community mandate. In this study, an active organization was defined as a registered NGO that was operational during the study period and had documented ongoing activities within its declared community mandate. The Directorate’s registry identified 13 NGOs that met these inclusion criteria; therefore, all eligible organizations were included through a census approach rather than selecting a subset. This yielded a final population of 13 organizations and 109 board members (*N* = 109), representing a 100% response rate. The distribution of respondents across organizations is presented in Table 1.

**Table 1:** Distribution of Board members across the study organizations (*N* = 109).

Index	Organization	*N*
1	Al-Mustaqbal Association for Development and Consumer Protection	13
2	Fatima Al-Zahraa Islamic Association for Community Development	7
3	Ahdaf wa Risala Association for Sustainable Development	13
4	Al-Keram Association for Community Development	13
5	Qur’an Preservation Association — Al-Sharawna	9
6	Ghaith Association for Women’s Education and Development	7
7	Isad Usra Faqira Association — Sabil Maki, Kom Ombo	11
8	Arab Association for Media, Development, and Training	7
9	Al-Samaha Association for Local Community Development	7
10	Community Development Association — Al-Daqadiq	5
11	Youth of the Future Association — Al-Radisiyya	5
12	Kheir Baladna Association	5
13	Comprehensive Development and Continuing Education Association	7
	**Total**	**109**

### 4.3 Data collection and analysis

Data were collected through a structured self-administered questionnaire developed specifically for this study, grounded in the relevant theoretical literature and informed by prior empirical studies on NGO organizational capacity and crisis preparedness. The questionnaire items were developed based on previous studies related to NGO organizational capacity and crisis preparedness, in addition to the theoretical framework guiding the study. The instrument was organized around four organizational requirements: administrative, human resource, financial, and legislative requirements. The preliminary version of the questionnaire was reviewed by five experts to assess the clarity, relevance, and alignment of the items with the study objectives, and necessary modifications were made before finalizing the instrument. The final instrument comprised 16 closed-ended items distributed across four organizational requirements (administrative, human resource, financial, and legislative requirements), with four items per requirement. Items were rated on a five-point Likert scale ranging from Strongly Agree (5) to Strongly Disagree (1). To assess internal consistency, Cronbach’s alpha for the overall instrument was 0.902, indicating excellent internal consistency. To classify the level of availability of each organizational requirement, a standardized interpretation grid was applied. The scale range (5–1 = 4) was divided by five equal intervals, yielding a cell width of 0.80. The resulting classification thresholds are presented in Table 2.

**Table 2:** Mean score interpretation grid.

Mean range	Level
1.00–1.80	Very low
1.81–2.60	Low
2.61–3.40	Moderate
3.41–4.20	High
4.21–5.00	Very high

Data were entered, coded, and analyzed using the Statistical Package for the Social Sciences (SPSS, Version 26). A multi-layered analytical strategy was employed to address the study’s research questions systematically. First, descriptive statistics including means (M) and standard deviations (SD) were computed for each item and each of the four organizational requirement categories. Item-level means were used to rank statements within each requirement category from highest to lowest, identifying which organizational requirements were most and least available. Mean scores for each requirement category were subsequently classified against the interpretation grid in Table 2. Second, a requirement-category ranking table was constructed, presenting the four organizational requirement categories in descending order of their mean scores, enabling an integrated assessment across the different organizational requirement categories and a clear identification of structural capacity gaps. Third, inferential statistics were applied to examine whether statistically significant differences existed in organizational requirements according to respondents’ personal and organizational characteristics. Specifically, independent samples t-tests were used for the dichotomous grouping variable of gender, while one-way Analysis of Variance (ANOVA) was employed for variables with three or more categories (age, educational qualification, organizational role, years of experience, and organizational age). Where ANOVA yielded significant results, post-hoc tests were conducted to identify between-group differences. A significance threshold of *p* < 0.05 was adopted throughout all inferential analyses. Fourth, Pearson correlation coefficients were computed to examine the relationships among the four organizational requirements. Finally, a multiple regression analysis was conducted to identify the relative contribution of organizational age, years of experience, and gender to the overall score of organizational requirements.

This study focused on environmentally engaged NGOs registered and operating in Aswan Governorate, Egypt, and examined their organizational and administrative requirements for responding to environmental crises. The study examined administrative, human resource, financial, and legislative requirements as indicators of NGO organizational capacity for crisis management. However, several limitations should be considered when interpreting the findings. First, the cross-sectional design captures organizational conditions at a single point in time and does not allow conclusions regarding changes in organizational capacity or crisis response capacity over time. Second, the study relied on self-reported responses from NGO board members and executive representatives, which may be influenced by individual perceptions. Third, although the census approach included all eligible organizations within the defined study setting, the findings may not be fully generalizable to NGOs operating in other geographical or institutional contexts.

Ethical approval for this study was obtained from the Research Ethics Committee, Hohai University (approval number: HHU-REC-2026-0142), and written informed consent was obtained from all participants prior to data collection.

## 5 Finding

### 5.1 Sample characteristics

A total of 109 board members from 13 environmentally engaged NGOs in Aswan Governorate participated in this study, representing a 100% response rate given the comprehensive census design. The demographic and organizational profile of participants is presented in Table 3. It should be noted that percentages for fields of operation and types of environmental crises do not sum to 100%, as respondents were permitted to select multiple options.

**Table 3:** Demographic and organizational characteristics of study participants (*N* = 109).

Variable	Category	*n*	%
Gender	Male	72	66.1
	Female	37	33.9
Age	Under 30 years	10	9.2
	30 to < 40 years	38	34.9
	40 to < 50 years	38	34.9
	50 years and above	23	21.0
Educational qualification	Intermediate qualification	13	12.0
	Above-intermediate qualification	18	16.5
	University degree	70	64.2
	Postgraduate degree	8	7.3
Role within NGO	Board Chair	13	11.9
	Deputy Board Chair	9	8.3
	Board Member	60	55.0
	Treasurer	10	9.2
	Secretary	10	9.2
	Executive Director	7	6.4
Years of experience	Less than 5 years	47	43.2
	5 to 10 years	42	38.5
	More than 10 years	20	18.3
Organizational age	Less than 5 years	23	21.1
	5 to 10 years	12	11.0
	11 to 20 years	42	38.5
	More than 20 years	32	29.4
Workforce size	Fewer than 10	12	11.0
	10 to 50	79	72.5
	51 to 200	7	6.4
	More than 200	11	10.1
Fields of operation*	Environment and Sustainable Development	90	82.6
	Relief and Disaster Management	49	45.0
	Health and Social Services	84	77.1
	Community Development	66	60.6
	Education and Awareness	81	74.3
Types of environmental crises faced or anticipated*	Floods/Flash floods	41	37.6
	Water scarcity/Drought	18	16.5
	Environmental pollution	87	79.8
	Fires	18	16.5
	Dust/Sandstorms	51	46.8
	Earthquakes/Natural disasters	15	13.8
Prior crisis response experience	Yes	63	57.8
	No	46	42.2
	**Total**	**109**	**100**

* Multiple responses permitted; percentages do not sum to 100%.

Regarding gender, male board members constituted the majority at 66.1%, compared to 33.9% female, reflecting the predominance of male representation in NGO governance structures within the study context. With respect to age, the largest proportions fell within the 30–39 and 40–49 age brackets (34.9% each), followed by those aged 50 and above (21.0%) and those under 30 (9.2%), indicating that the studied NGOs rely predominantly on middle-aged professionals in their governance structures. Concerning educational attainment, the majority held a university degree (64.2%), followed by above-intermediate (16.5%), intermediate (12.0%), and postgraduate qualifications (7.3%), suggesting a relatively high level of formal education among board members. In terms of organizational role, more than half served as general board members (55.0%), with the remainder distributed across board chair (11.9%), treasurer and secretary (9.2% each), deputy chair (8.3%), and executive director (6.4%). Regarding years of experience, 43.2% had fewer than five years of tenure within their organization, 38.5% between five and ten years, and 18.3% more than ten years, reflecting relatively recent organizational tenure for a substantial portion of respondents. As for organizational age, the largest share of NGOs had been operating for 11 to 20 years (38.5%), followed by those established for more than 20 years (29.4%), those under five years (21.1%), and those between five and ten years (11.0%), reflecting a predominantly established organizational landscape. With regard to workforce size, the vast majority of NGOs employed between 10 and 50 staff and volunteers (72.5%), with smaller proportions employing fewer than 10 (11.0%), more than 200 (10.1%), or between 51 and 200 personnel (6.4%). Regarding fields of operation, the most prevalent were environment and sustainable development (82.6%), followed by health and social services (77.1%), education and awareness (74.3%), community development (60.6%), and relief and disaster management (45.0%). The high prevalence of multi-sector engagement reflects the integrated operational mandates of the studied organizations. With respect to types of environmental crises faced or anticipated, environmental pollution was the most commonly reported (79.8%), followed by dust and sandstorms (46.8%), floods and flash floods (37.6%), water scarcity (16.5%), fires (16.5%), and earthquakes or natural disasters (13.8%), reflecting the specific environmental risk profile of Aswan Governorate. Finally, concerning prior crisis response experience, 57.8% of participating organizations had previously engaged in responding to an environmental crisis, while 42.2% had not, indicating that a majority of the studied NGOs possess some degree of practical crisis management experience.

### 5.2 Organizational requirements of NGOs in facing environmental crises

#### 5.2.1 Administrative requirements

Table 4 shows that the sample’s level of administrative requirements for NGOs in facing environmental crises was very high, with a mean score of 4.06. According to the ranking of mean scores, first came the availability of flexible internal bylaws enabling rapid decision-making without lengthy routine procedures (M = 4.50), followed by the NGO’s possession of a clear organizational structure defining authorities and responsibilities during environmental crises (M = 4.35), and finally the NGO’s possession of written, updated emergency plans for managing environmental crises (M = 3.40).

**Table 4:** Descriptive statistics for administrative requirements.

Statement	Strongly agree *n*(%)	Agree *n*(%)	Neutral *n*(%)	Disagree *n*(%)	Strongly disagree *n*(%)	Mean	SD	Rank
The NGO has a clear organizational structure that defines authorities and responsibilities during environmental crises	38 (34.9%)	71 (65.1%)	-	-	-	4.35	0.47	2
Flexible internal bylaws are available that allow rapid decision-making without lengthy routine procedures	56 (51.4%)	52 (47.7%)	1 (0.9%)	-	-	4.50	0.52	1
The NGO has written and updated emergency plans specific to environmental crisis management	8 (7.3%)	42 (38.5%)	45 (41.3%)	14 (12.8%)	-	3.40	0.80	4
Internal coordination between the NGO’s departments and units occurs effectively during environmental crises	14 (12.8%)	77 (70.6%)	18 (16.5%)	-	-	3.96	0.54	3
					**4.06**	**0.67**	**High**	

Bold values indicate the mean and standard deviation for the requirement as a whole.

It is noted that although administrative requirements as a whole reached a high level, the item concerning written and updated emergency plans specifically for environmental crisis management received the lowest mean score within this dimension.

#### 5.2.2 Human resource requirements

Table 5 shows that the sample’s level of human resource requirements for NGOs in facing environmental crises was high, with a mean score of 3.80. According to the ranking of mean scores, first came the prevalence of team spirit and high motivation among the NGO’s personnel when facing environmental crises (M = 4.17), followed by the NGO’s possession of human resources qualified and trained to deal with environmental crises (M = 3.80), and finally the provision of periodic training to staff and volunteers in crisis management, environmental work, and first aid (M = 3.56).

**Table 5:** Descriptive statistics for human resource requirements.

Statement	Strongly agree *n*(%)	Agree *n*(%)	Neutral *n*(%)	Disagree *n*(%)	Strongly disagree *n*(%)	Mean	SD	Rank
The NGO has human resources qualified and trained to deal with environmental crises	11 (10.1%)	65 (59.6%)	33 (30.3%)	-	-	3.80	0.60	2
Staff and volunteers receive periodic training in crisis management, environmental work, and first aid	10 (9.2%)	49 (45.0%)	42 (38.5%)	8 (7.3%)	-	3.56	0.76	4
Staff members’ functional roles during crises are clear, avoiding duplication and conflict	6 (5.5%)	72 (66.1%)	19 (17.4%)	12 (11.0%)	-	3.66	0.75	3
A spirit of teamwork and high motivation prevails among the NGO’s personnel when facing environmental crises	19 (17.4%)	90 (82.6%)	-	-	-	4.17	0.38	1
					**3.80**	**0.65**	**High**	

Bold values indicate the mean and standard deviation for the requirement as a whole.

#### 5.2.3 Financial requirements

Table 6 shows that the sample’s level of financial requirements for NGOs in facing environmental crises was high, with a mean score of 3.75. According to the ranking of mean scores, first came the NGO’s adherence to principles of transparency and financial accountability toward funders and supporters (M = 4.56), followed by the NGO’s reliance on diverse funding sources not limited to a single donor or source (M = 3.88), and finally the NGO’s possession of a financial reserve dedicated to emergencies and environmental crisis response (M = 3.07).

**Table 6:** Descriptive Statistics for Financial Requirements.

Statement	Strongly agree *n*(%)	Agree *n*(%)	Neutral *n*(%)	Disagree *n*(%)	Strongly disagree *n*(%)	Mean	SD	Rank
The NGO relies on diverse funding sources and is not limited to a single donor or source	20 (18.3%)	69 (63.3%)	7 (6.4%)	13 (11.9%)	-	3.88	0.84	2
The NGO has a financial reserve dedicated to emergencies and responding to environmental crises	7 (6.4%)	38 (34.9%)	33 (30.3%)	18 (16.5%)	13 (11.9%)	3.07	1.11	4
Financial resources are efficiently managed and expenditures rationalized appropriately during crises	15 (13.8%)	52 (47.7%)	11 (10.1%)	31 (28.4%)	-	3.47	1.05	3
The NGO adheres to principles of transparency and financial accountability toward funders and supporters	61 (56.0%)	48 (44.0%)	-	-	-	4.56	0.50	1
					**3.75**	**0.65**	**High**	

Bold values indicate the mean and standard deviation for the requirement as a whole.

This variability may partly reflect differences in donor diversification across the sampled organizations.

#### 5.2.4 Legislative requirements

Table 7 shows that the sample’s level of legislative requirements for NGOs in facing environmental crises was high, with a mean score of 3.77. According to the ranking of mean scores, first came the NGO’s compliance with the laws and regulations governing civil society work in the field of environmental crises (M = 4.27), followed by the clarity and effectiveness of cooperation mechanisms with government bodies concerned with the environment during crises (M = 3.76), and finally the adequacy of legal protection afforded to staff and volunteers intervening in environmental crises (M = 3.29).

**Table 7:** Descriptive statistics for legislative requirements.

Statement	Strongly agree *n*(%)	Agree *n*(%)	Neutral *n*(%)	Disagree *n*(%)	Strongly disagree *n*(%)	Mean	SD	Rank
The NGO complies with the laws and regulations governing civil society work in the field of environmental crises	36 (33.0%)	66 (60.6%)	7 (6.4%)	-	-	4.27	0.57	1
Cooperation mechanisms with government bodies concerned with the environment are clear and effective during crises	26 (23.9%)	44 (40.4%)	26 (23.9%)	13 (11.9%)	-	3.76	0.95	2
Current legislation allows the NGO to act quickly and take necessary measures in emergencies	11 (10.1%)	72 (66.1%)	13 (11.9%)	13 (11.9%)	-	3.74	0.80	3
Staff and volunteers enjoy adequate legal protection while intervening in environmental crises	14 (12.8%)	39 (35.8%)	32 (29.4%)	13 (11.9%)	11 (10.1%)	3.29	1.15	4
					**3.77**	**0.65**	**High**	

Bold values indicate the mean and standard deviation for the requirement as a whole.

#### 5.2.5 Comparative ranking of organizational requirements

Table 8 shows that the sample’s overall level of organizational requirements for NGOs in facing environmental crises was high, with a mean score of 3.84. According to the ranking of mean scores for each requirement individually, administrative requirements ranked first (M = 4.06), followed by human resource requirements (M = 3.80), then legislative requirements (M = 3.77), and finally financial requirements (M = 3.75). Figure 1 illustrates the mean scores of the four organizational requirements relative to the high-level threshold.

**Table 8:** Ranking of the four organizational requirements.

Index	Requirement	Mean	SD	Level	Rank
1	Administrative Requirements	4.06	0.67	Very High	1
2	Human Resource Requirements	3.80	0.65	High	2
3	Legislative Requirements	3.77	0.65	High	3
4	Financial Requirements	3.75	0.65	High	4
**Organizational requirements overall**		**3.84**	**0.49**	**High**	

Bold values indicate the mean and standard deviation for the requirement as a whole.

**Figure 1:**
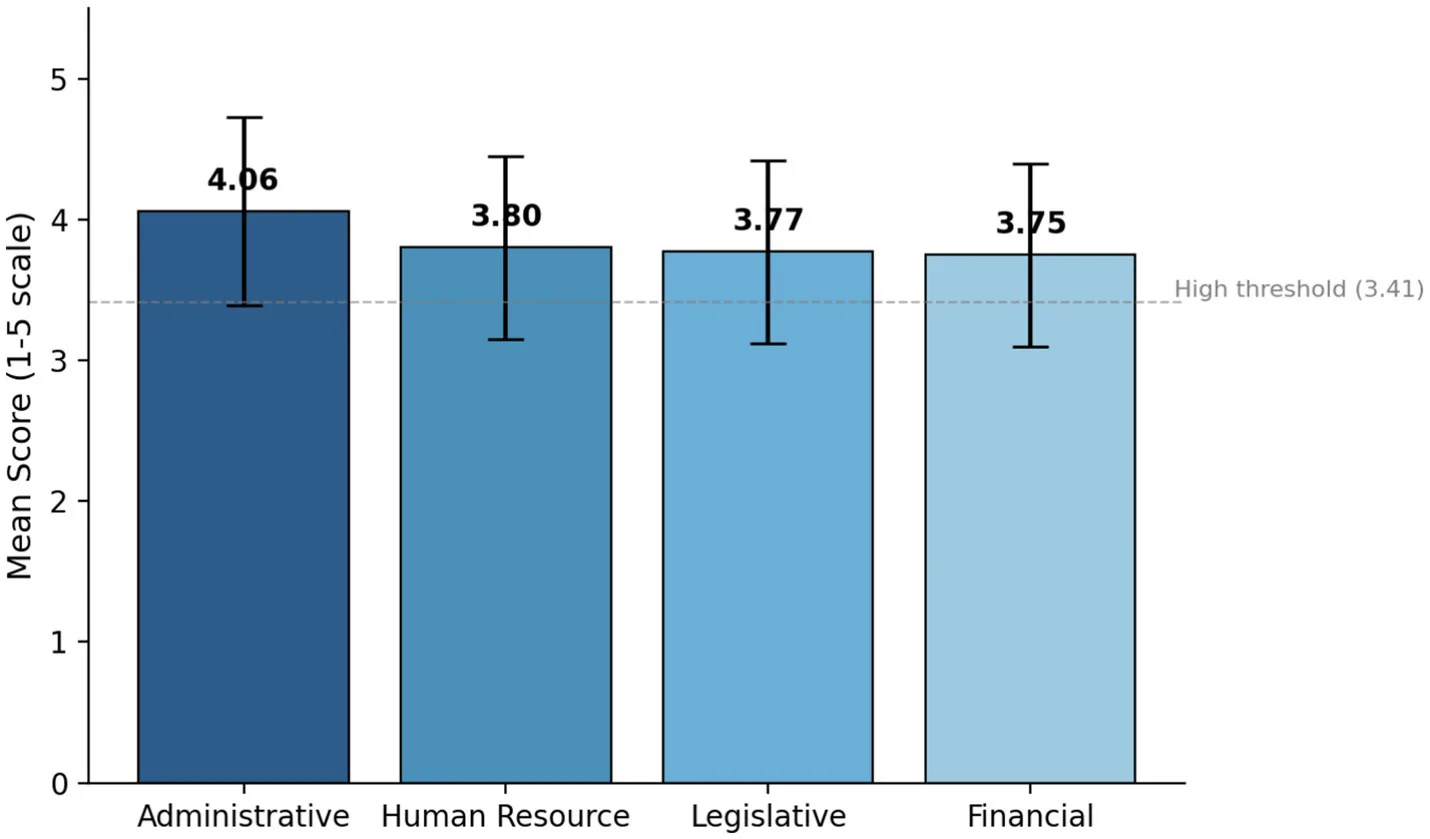
Mean scores of the four organizational requirements.

### 5.3 Differences in organizational requirements according to personal and organizational variables

#### 5.3.1 Differences according to gender

Table 9 shows statistically significant differences between male and female respondents in some organizational requirements. Significant differences emerged in the administrative and human resource requirements: the calculated t values were 2.35 and 1.98, respectively, both exceeding the critical t value of 1.96 at the 0.05 level, with significance levels of 0.005 and 0.040, respectively, both below the adopted threshold. These differences favored male respondents, whose mean scores were higher than those of female respondents. By contrast, no statistically significant differences emerged in the financial and legislative requirements: the calculated t values were 0.63 and 1.07, respectively, both below the critical value, with significance levels of 0.892 and 0.470, both above the 0.05 level, indicating convergence between male and female responses in these two requirements. Accordingly, the null hypothesis is partially rejected and the alternative hypothesis partially accepted: differences according to gender exist in some organizational requirements (administrative and human resource), while no differences exist in the financial and legislative requirements.

**Table 9:** Independent samples t-test by gender.

Dimension	Male (*n* = 72) M	Male SD	Female (*n* = 37) M	Female SD	t	*p*
Administrative requirements	16.42	1.54	15.84	1.01	2.35	0.005
Human resource requirements	15.40	1.94	14.78	1.29	1.98	0.040
Financial requirements	15.11	3.14	14.72	2.95	0.63	0.892
Legislative requirements	15.25	2.70	14.70	2.44	1.07	0.470

df = 107. Critical t = 1.96 at the 0.05 level and 2.58 at the 0.01 level.

#### 5.3.2 Differences according to age

Table 10 shows that the one-way ANOVA results for the age variable revealed a statistically significant difference in the administrative requirements, but no statistically significant differences in the remaining requirements or the overall score. The calculated *F* values for the administrative, human resource, financial, and legislative requirements, and the overall score, were 2.76, 1.20, 0.85, 1.48, and 0.93, respectively, with corresponding significance levels of 0.046, 0.321, 0.471, 0.226, and 0.429. The significance level for administrative requirements (0.046) falls below the adopted 0.05 threshold, indicating a statistically significant effect of age on this requirement specifically, whereas the remaining significance levels all exceed 0.05, indicating no statistically significant effect of age on the other requirements or the overall score. Accordingly, the null hypothesis stating that “age has no effect on the organizational requirements of NGOs in facing environmental crises” is rejected with respect to administrative requirements, and accepted with respect to the remaining requirements and the overall score.

**Table 10:** One-way ANOVA by age.

Requirement	Source	SS	df	MS	F	*p*
Administrative requirements	Between groups	13.26	3	4.42	2.76	0.046
	Within groups	168.39	105	1.60		
	Total	181.65	108			
Human resource requirements	Between groups	11.68	3	3.89	1.20	0.321
	Within groups	339.60	105	3.23		
	Total	351.27	108			
Financial requirements	Between groups	23.74	3	7.91	0.85	0.471
	Within groups	979.71	105	9.33		
	Total	1,003.45	108			
Legislative requirements	Between groups	28.04	3	9.35	1.48	0.226
	Within groups	665.51	105	6.34		
	Total	693.56	108			
Organizational requirements overall	Between groups	695.29	3	231.76	0.93	0.429
	Within groups	26,141.85	105	248.97		
	Total	26,837.14	108			

Critical F = 2.45 at the 0.05 level and 3.48 at the 0.01 level. df between = 3, df within = 105.

#### 5.3.3 Differences according to educational qualification

Table 11 shows that the one-way ANOVA results for the educational qualification variable revealed a statistically significant difference in the financial requirements, but no statistically significant differences in the remaining requirements or the overall score. The calculated *F* values for the administrative, human resource, financial, and legislative requirements, and the overall score, were 0.45, 0.33, 3.449, 1.834, and 2.67, respectively, with corresponding significance levels of 0.715, 0.801, 0.019, 0.145, and 0.051. The *F* value for financial requirements (3.449) exceeds the tabulated critical *F* value (2.45) at the 0.05 level, with an exact significance level of 0.019, indicating a statistically significant effect of educational qualification on this requirement. Although the *F* value for the overall score (2.67) also exceeds the critical value, its exact significance level (0.051) narrowly exceeds the conventional 0.05 threshold, and this measure is therefore treated as not statistically significant. No statistically significant effect of educational qualification was found on the administrative, human resource, or legislative requirements. Accordingly, the null hypothesis stating that “educational qualification has no effect on the organizational requirements of NGOs in facing environmental crises” is rejected with respect to financial requirements only, and accepted with respect to the remaining requirements and the overall score.

**Table 11:** One-way ANOVA by educational qualification.

Requirements	Source	SS	df	MS	F	*p*
Administrative requirements	Between groups	2.72	3	0.91	0.45	0.715
	Within groups	209.99	105	2.00		
	Total	212.72	108			
Human resource requirements	Between groups	3.31	3	1.10	0.33	0.801
	Within groups	346.83	105	3.30		
	Total	350.15	108			
Financial requirements	Between groups	90.50	3	30.17	3.449	0.019
	Within groups	918.27	105	8.75		
	Total	1,008.77	108			
Legislative requirements	Between groups	32.12	3	10.71	1.834	0.145
	Within groups	612.84	105	5.84		
	Total	644.95	108			
Organizational requirements overall	Between groups	1,926.42	3	642.14	2.67	0.051
	Within groups	25,275.69	105	240.72		
	Total	27,202.11	108			

Critical F = 2.45 at the 0.05 level and 3.48 at the 0.01 level. df between = 3, df within = 105.

#### 5.3.4 Differences according to role within the NGO

Table 12 shows that the one-way ANOVA results for the role variable revealed no statistically significant differences among sample members across any of the organizational requirements or the overall score. The calculated *F* values for the administrative, human resource, financial, and legislative requirements, and the overall score, were 0.78, 0.53, 0.57, 0.97, and 0.63, respectively, with corresponding significance levels of 0.570, 0.753, 0.720, 0.442, and 0.679, all above the adopted 0.05 level, indicating no statistically significant effect of role on organizational requirements across their various requirements or the overall score. This reflects convergence in sample members’ responses toward the organizational requirements necessary for facing environmental crises, regardless of their differing roles, indicating a degree of agreement in the perception of these requirements across different functional categories within the NGOs. Accordingly, the null hypothesis stating that “role has no effect on the organizational requirements of NGOs in facing environmental crises” is accepted, and the alternative hypothesis is rejected.

**Table 12:** One-way ANOVA by role.

Requirements	Source	SS	df	MS	F	*p*
Administrative requirements	Between groups	7.72	5	1.54	0.78	0.570
	Within Groups	205.00	103	1.99		
	Total	212.72	108			
Human resource requirements	Between Groups	8.44	5	1.69	0.53	0.753
	Within Groups	328.51	103	3.19		
	Total	336.95	108			
Financial requirements	Between Groups	27.59	5	5.52	0.57	0.720
	Within Groups	990.37	103	9.62		
	Total	1,017.96	108			
Legislative requirements	Between Groups	33.08	5	6.62	0.97	0.442
	Within Groups	705.48	103	6.85		
	Total	738.55	108			
Organizational requirements overall	Between Groups	809.26	5	161.85	0.63	0.679
	Within Groups	26,542.01	103	257.69		
	Total	27,351.27	108			

df between = 5, df within = 103.

#### 5.3.5 Differences according to years of experience within the organization

Table 13 shows that the one-way ANOVA results for the years-of-experience variable revealed statistically significant differences in the administrative, financial, and legislative requirements, as well as the overall score, but no statistically significant difference in the human resource requirement. The calculated *F* values for the administrative, human resource, financial, and legislative requirements, and the overall score, were 9.138, 2.684, 4.149, 5.520, and 6.698, respectively, with corresponding significance levels of 0.000, 0.073, 0.018, 0.005, and 0.002. With the exception of the human resource requirement (*p* = 0.073, above the 0.05 threshold), all significance levels fall below 0.05, indicating a statistically significant effect of years of experience on most organizational requirements. This suggests that occupational experience is an influential factor in shaping sample members’ perceptions of organizational requirements, with the effect being particularly pronounced for administrative requirements (*p* < 0.001). Accordingly, the null hypothesis stating that “years of experience have no effect on the organizational requirements of NGOs in facing environmental crises” is rejected with respect to the administrative, financial, and legislative requirements and the overall score, and accepted with respect to the human resource requirement.

**Table 13:** One-way ANOVA by years of experience.

Requirements	Source	SS	df	MS	F	*p*
Administrative requirements	Between groups	31.28	2	15.64	9.138	000
	Within groups	181.43	106	1.71		
	Total	212.72	108			
Human resource requirements	Between groups	16.24	2	8.12	0.684	0.073
	Within groups	320.72	106	3.03		
	Total	336.95	108			
Financial requirements	Between groups	73.90	2	36.95	4.149	0.018
	Within groups	944.06	106	8.91		
	Total	1,017.96	108			
Legislative requirements	Between groups	69.67	2	34.84	5.520	0.005
	Within groups	668.88	106	6.31		
	Total	738.55	108			
Organizational requirements overall	Between groups	3,125.17	2	1562.58	**6.698**	0.002
	Within groups	24,730.41	106	233.31		
	Total	27,855.58	108			

df between = 2, df within = 106. Bold values indicate statistically significant results (*p* < 0.05).

#### 5.3.6 Differences according to organizational age

Table 14 shows that the one-way ANOVA results for the organizational age variable revealed statistically significant differences among sample members across all organizational requirements for NGOs in facing environmental crises, as well as in the overall score. The calculated *F* values for the administrative, human resource, financial, and legislative requirements, and the overall score, were 26.42, 10.17, 18.55, 20.14, and 24.04, respectively, with corresponding significance levels of 0.000 for all, below the adopted 0.05 level, indicating a statistically significant effect of organizational age on the different organizational requirements and the overall score. This indicates that sample members’ assessments of organizational requirements differ according to the NGO’s organizational age, which may reflect differences in organizational experience, institutional accumulation, and the level of administrative and operational development between newer and older NGOs, thereby affecting their perception of the requirements necessary for facing environmental crises. Accordingly, the null hypothesis stating that “organizational age has no effect on the organizational requirements of NGOs” is rejected. Because the overall F-test was significant, Tukey HSD post-hoc comparisons were conducted to identify which specific age groups differed from one another; these are presented in Table 15.

**Table 14:** One-way ANOVA by organizational age.

Requirements	Source	SS	df	MS	F	*p*
Administrative Requirements	Between groups	91.49	3	30.50	26.42	< 0.001
	Within groups	121.22	105	1.16		
	Total	212.72	108			
Human Resource Requirements	Between groups	75.84	3	25.28	10.17	< 0.001
	Within groups	261.12	105	2.49		
	Total	336.95	108			
Financial Requirements	Between groups	352.56	3	117.52	18.55	< 0.001
	Within groups	665.40	105	6.34		
	Total	1,017.96	108			
Legislative Requirements	Between groups	269.79	3	89.93	20.14	< 0.001
	Within groups	468.76	105	4.46		
	Total	738.55	108			
Organizational Requirements Overall	Between groups	11,965.19	3	3988.40	24.04	< 0.001
	Within groups	17,418.63	105	165.89		
	Total	29,383.82	108			

df between = 3, df within = 105. Organizational age groups: 1 = less than 5 years, 2 = 5 to 10 years, 3 = 11 to 20 years, 4 = more than 20 years.

**Table 15:** Tukey HSD post-hoc comparisons for organizational age.

Requirements	Group comparison	Mean difference	*p*
Administrative requirements	Group 1 vs. Group 4	2.190*	< 0.001
	Group 2 vs. Group 4	2.125*	< 0.001
	Group 3 vs. Group 4	1.839*	< 0.001
Human resource requirements	Group 1 vs. Group 4	1.481*	< 0.001
	Group 2 vs. Group 4	1.438*	< 0.001
	Group 3 vs. Group 4	2.009*	< 0.001
Financial requirements	Group 1 vs. Group 4	5.019*	< 0.001
	Group 2 vs. Group 4	3.063*	< 0.001
	Group 3 vs. Group 4	1.777*	< 0.001
Legislative requirements	Group 1 vs. Group 4	4.141*	< 0.001
	Group 2 vs. Group 4	3.250*	< 0.001
	Group 3 vs. Group 4	1.179*	< 0.001
Organizational requirements overall	Group 1 vs. Group 4	29.185*	< 0.001
	Group 2 vs. Group 4	19.750*	< 0.001
	Group 3 vs. Group 4	13.060*	< 0.001

#### 5.3.7 Post-hoc comparisons for organizational age

Table 15, presenting the Tukey HSD post-hoc comparison results for the organizational age variable, shows statistically significant differences between certain groups in favor of Group 4. The results show statistically significant differences between Group 4 and each of Groups 1, 2, and 3 across all organizational requirements (administrative, human resource, financial, and legislative), as well as the overall score, with significance levels of 0.000 in all comparisons. This indicates that the oldest NGOs (Group 4, more than 20 years) differ substantially from the other groups in their assessment of the organizational requirements necessary for facing environmental crises. These results further indicate that organizational age is an influential variable in shaping sample members’ perception of the level of organizational requirements, with responses varying according to organizational age, reflecting the role of accumulated experience or institutional recency in determining the level of awareness of organizational requirements within NGOs.

### 5.4 Correlation and regression analysis

#### 5.4.1 Correlation analysis among the four requirements

Table 16 shows that all four Requirements were significantly and positively correlated with one another (all *p* < 0.001). The strongest correlation was observed between financial and legislative requirements (r = 0.926), while the weakest was between human resource and legislative requirements (r = 0.533). Correlations among the remaining pairs ranged from r = 0.631 to r = 0.710. This pattern is illustrated in Figure 2.

**Table 16:** Correlations among the four requirements.

Requirements	Administrative	Human resource	Financial	Legislative
Administrative	1.00			
Human resource	0.648**	1.00		
Financial	0.710**	0.631**	1.00	
Legislative	0.670**	0.533**	0.926**	1.00

** *p* < 0.001 for all correlations.

**Figure 2:**
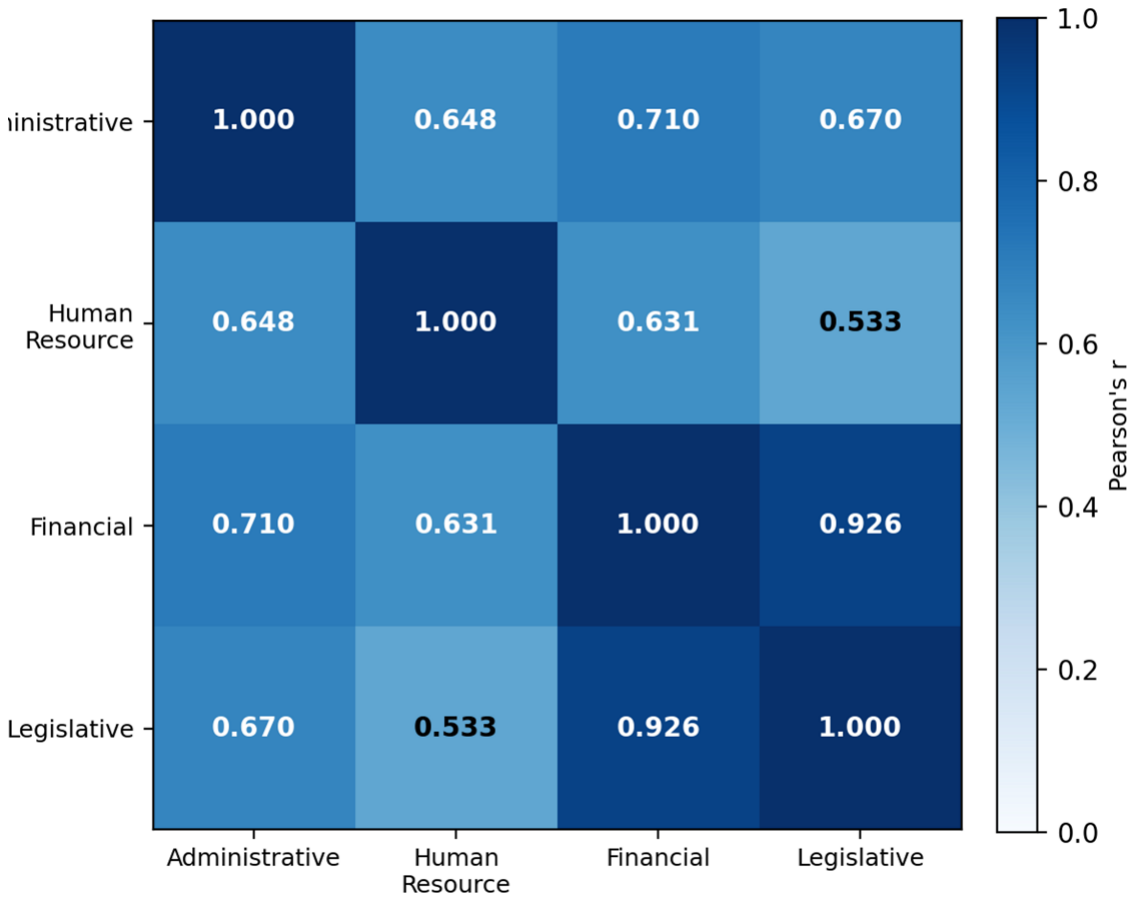
Correlation matrix among the four organizational requirements.

#### 5.4.2 Multiple regression predicting overall organizational requirements

Table 17 shows that when organizational age, years of experience, and gender were entered simultaneously into a multiple regression predicting overall organizational requirements, the model was statistically significant, F(3, 105) = 19.38, *p* < 0.001, explaining 35.6% of the variance (R^2^ = 0.356). Organizational age was a significant independent predictor (B = 3.86, *p* < 0.001), whereas years of experience (B = 1.37, *p* = 0.133) and gender (B = −0.70, *p* = 0.604) were not.

**Table 17:** Multiple regression predicting overall organizational requirements.

Predictor	B	SE	t	*p*
Constant	49.36	2.98	16.58	< 0.001
Organizational Age	3.86	0.59	6.51	< 0.001
Years of Experience	1.37	0.90	1.52	0.133
Gender	−0.70	1.35	−0.52	0.604

R^2^ = 0.356, Adjusted R^2^ = 0.338, F(3, 105) = 19.38, *p* < 0.001.

## 6 Discussion

This study examined the organizational and administrative requirements of NGOs responding to environmental crises in Aswan, Egypt. The overall level of organizational requirements was high (M = 3.84), with administrative requirements ranking highest (M = 4.06) and financial requirements ranking lowest (M = 3.75). The four organizational requirements were positively correlated with one another. Gender, years of experience, and organizational age were significantly associated with organizational requirements, with organizational age emerging as the strongest predictor. These findings are discussed below in relation to theory and prior literature. Administrative requirements emerged as the most developed capacity among the studied NGOs, whereas financial requirements, despite remaining within the high range overall, showed the greatest internal variation, combining the study’s highest-rated item (financial transparency) with its lowest-rated (emergency financial reserves). The four organizational requirements were also found to be significantly and positively correlated with one another, with an especially strong association between financial and legislative requirements (r = 0.926), suggesting that these two requirements may be shaped by closely related organizational conditions rather than developing independently. This pattern of results is only partly consistent with prior literature. The gap between administrative structure and financial contingency planning mirrors findings from outside the region: in a survey of more than 600 U.S. nonprofits conducted at the onset of the COVID-19 pandemic, Kim and Mason (75) found that organizations holding larger operating reserves were significantly less likely to cut operating hours or lose staff once the crisis hit, while reserve levels varied widely even among otherwise well-managed organizations. Using data on nonprofits affected by Hurricane Sandy, Chen (76) similarly found that post-disaster financial distress was driven largely by structural characteristics rather than by general administrative quality. Both studies therefore agree with the present finding that financial contingency planning is a distinct capacity that does not automatically follow from strong administrative practice.

The strong correlation between financial and legislative requirements, however, has not been extensively documented in prior NGO capacity research, which has more commonly treated financial management and regulatory compliance as conceptually and empirically distinct areas. In this respect, the present finding diverges from and extends the existing literature rather than simply confirming it.

Organization Theory offers a plausible explanation for both patterns. The divergence between administrative and financial capacity is consistent with the resource-dependence perspective, which frames financial capacity as contingent on external funding relationships rather than on internal structure alone. The close coupling between financial and legislative requirements, meanwhile, is consistent with Organization Theory’s institutional perspective: both requirements may be jointly shaped by an NGO’s standing with the same external audiences, government regulators and funders such that legislative compliance and financial transparency function as parallel signals of institutional legitimacy rather than as independent operational tasks.

With regard to the second research question, statistically significant differences in organizational requirements were found according to some, but not all, of the examined characteristics. Male respondents reported significantly higher administrative and human resource requirements than female respondents, t(107) = 2.35, *p* = 0.005, and t(107) = 1.98, *p* = 0.040, respectively, with no significant gender differences in the financial or legislative requirements. Age, educational qualification, and organizational role showed little consistent association with organizational requirements: age was linked only to the administrative requirement, *F* (3, 105) = 2.76, *p* = 0.046; educational qualification was linked only to financial requirements, *F* (3, 105) = 3.45, *p* = 0.019; and role showed no significant association with any dimension. By contrast, years of organizational experience were significantly associated with four of the five measures examined, *F* (2, 106) ranging from 4.15 to 9.14, all *p* < 0.05, with the human resource dimension the only exception. Organizational age produced the most consistent pattern in the study, with significant differences across all four requirements and the overall score, *F* (3, 105) ranging from 10.17 to 26.42, all *p* < 0.001; Tukey HSD comparisons showed that NGOs older than twenty years scored significantly higher than each younger group, with no significant differences among the younger groups themselves. Finally, a multiple regression entered organizational age, years of experience, and gender simultaneously found that organizational age remained the only significant independent predictor of overall organizational requirements score, B = 3.86, *p* < 0.001, explaining 35.6% of the variance, while years of experience and gender did not retain significance once organizational age was accounted for.

These patterns only partly align with prior research. The male advantage found here contrasts with nonprofit evidence linking female leadership to stronger board performance and higher public service motivation (77, 78). The limited effect of age, qualification, and role echoes studies where organizational-level factors outweigh individual characteristics. The tenure effect is less consistent with prior work: some studies report a curvilinear tenure–attitude relationship (79), others no effect at all (80). The dominance of organizational age, however, matches the broader literature on institutional maturity as the more durable driver of organizational capacity, consistent with Alam et al. (81) finding that disaster-response capacity in coastal Bangladesh accumulated over time through the evolving contributions of households, NGOs, and the government.

Organization Theory helps explain this overall pattern: structural properties like administrative routines and regulatory relationships are encoded in the organization’s formal rules rather than in individual attributes, which is why most individual-level characteristics showed limited effects. The dominance of organizational age reflects the theory’s emphasis on accumulated structure and institutional memory. Older organizations have simply had more time to develop the routines this study measures, which also explains why organizational age outweighed individual tenure once both were modeled together. While this study primarily applies Organization Theory, its findings raise a question the theory does not directly address: why administrative and financial capacities would develop at different rates within the same organization. This suggests a potential refinement that different components of organizational capacity may be governed by different sub-mechanisms within Organization Theory (bureaucratic structure versus resource dependence) rather than developing as a single, unified capacity, as much of the classical literature implicitly assumes.

Several limitations should be noted. The sample of thirteen NGOs in a single governorate limits generalizability, the cross-sectional design cannot establish causality, and reliance on self-reported perceptions may be subject to social desirability bias.

## 7 Conclusion

This study examined the organizational and administrative requirements of NGOs responding to environmental crises in Aswan, Egypt, and found an overall high level of organizational requirements, led by administrative requirements and followed by financial requirements. Organizational age emerged as the strongest and most consistent predictor of the overall organizational requirements score, outweighing individual characteristics such as years of experience once both were considered together, underscoring institutional maturity as an important factor shaping organizational capacity in this context. These findings also contribute to Weber’s theory of bureaucracy by demonstrating that its core emphasis on formal structures, rule-governed procedures, and clear authority relationships remains empirically relevant within contemporary civil society organizations operating under crisis conditions. In particular, the finding that administrative requirements represented the most consistently developed aspect of organizational capacity among the studied NGOs, while financial requirements showed greater limitations, suggests that bureaucratic principles may be more readily established within internal organizational structures than in resource-dependent requirements influenced by external funding conditions. This extends Weberian perspectives by highlighting how bureaucratic features continue to shape organizational capacity beyond traditional state institutions, particularly in nonprofit organizations operating under conditions of uncertainty and resource constraints.

These findings suggest several practical directions for strengthening NGO capacity for crisis response. First, NGOs, particularly newer organizations, would benefit from developing dedicated financial reserves for emergencies, given that this was the weakest indicator across the entire study despite otherwise strong financial transparency. Second, formal, written crisis-management plans should be prioritized alongside the flexible administrative structures NGOs already report, since documented procedures cannot be assumed to follow from general administrative capacity alone. Third, given the pronounced effect of organizational age, capacity-building initiatives and donor support should specifically target younger NGOs to help accelerate the institutional development that older organizations have accumulated gradually. Finally, government bodies and funding agencies engaging with the NGO sector may consider the organizational age of prospective partners when assessing crisis-response readiness, while also investing in mechanisms, such as mentorship links between older and newer NGOs, that could more directly transfer accumulated institutional knowledge.

## Data Availability

The de-identified dataset is not publicly available due to the confidentiality assurances given to participants. Aggregated data may be made available by the corresponding author upon reasonable request.
