# Correction: Organizational and administrative requirements of NGOs for environmental crisis response: a field study of civil associations

**DOI:** 10.3389/fpubh.2026.2023790

**Published:** 2026-10-22

**Authors:** Frontiers Production Office

**Affiliations:** Frontiers Media SA, Lausanne, Switzerland

**Keywords:** civil society organizations, crisis management, Egypt, environmental crisis response, NGO capacity, organization theory

The Affiliation Universitas Islam Bandung for the reviewer Siska Nia Irasanti was erroneously given as Universitas Padjadjaran.

The original version of this article has been updated.

